# Applying ecological resistance and resilience to dissect bacterial antibiotic responses

**DOI:** 10.1126/sciadv.aau1873

**Published:** 2018-12-05

**Authors:** Hannah R. Meredith, Virgile Andreani, Helena R. Ma, Allison J. Lopatkin, Anna J. Lee, Deverick J. Anderson, Gregory Batt, Lingchong You

**Affiliations:** 1 Department of Biomedical Engineering, Duke University, Durham, NC, USA.; 2 Inria Saclay–Île-de-France, Palaiseau, France.; 3 Institut Pasteur, Paris, France.; 4 Division of Infectious Diseases, Department of Medicine, Duke University School of Medicine, Durham, NC, USA.; 5 Duke Center for Antimicrobial Stewardship and Infection Prevention, Duke University School of Medicine, Durham, NC, USA.; 6 Center for Genomic and Computational Biology, Duke University, Durham, NC, USA.; 7 Department of Molecular Genetics and Microbiology, Duke University School of Medicine, Durham, NC, USA.

## Abstract

An essential property of microbial communities is the ability to survive a disturbance. Survival can be achieved through resistance, the ability to absorb effects of a disturbance without a notable change, or resilience, the ability to recover after being perturbed by a disturbance. These concepts have long been applied to the analysis of ecological systems, although their interpretations are often subject to debate. Here, we show that this framework readily lends itself to the dissection of the bacterial response to antibiotic treatment, where both terms can be unambiguously defined. The ability to tolerate the antibiotic treatment in the short term corresponds to resistance, which primarily depends on traits associated with individual cells. In contrast, the ability to recover after being perturbed by an antibiotic corresponds to resilience, which primarily depends on traits associated with the population. This framework effectively reveals the phenotypic signatures of bacterial pathogens expressing extended-spectrum β-lactamases (ESBLs) when treated by a β-lactam antibiotic. Our analysis has implications for optimizing treatment of these pathogens using a combination of a β-lactam and a β-lactamase (Bla) inhibitor. In particular, our results underscore the need to dynamically optimize combination treatments based on the quantitative features of the bacterial response to the antibiotic or the Bla inhibitor.

## Teaser

Applying an ecological framework to bacterial antibiotic responses improves understanding of population recovery.

## INTRODUCTION

A disturbance is a biological, chemical, or physical event that affects a community (*1*). Given that the environment is constantly changing, an essential property of a community is its ability to recover after being disturbed. Responses to a disturbance include resistance, the ability to withstand perturbation in the presence of a disturbance; resilience, the ability to recover after being perturbed by a disturbance; or sensitivity, the inability to withstand or recover from a disturbance (*2*, *3*). Resistance and resilience have been documented in a range of systems and are often determined by different processes (*1*, *2*, *4*). Specifically, resistance is associated with processes that enable the tolerance of, or adaptation to, a disturbance, whereas resilience is associated with recolonization, reproduction, or rapid regrowth (*2*). The ability to identify the determinants for resistance versus resilience is crucial for predicting how a given community will respond to a disturbance as well as for designing strategies that will either preserve, change, or eliminate it (*3*, *5*). Although resistance and resilience have been defined in the literature for decades (*6*), the resistance-resilience framework has not been widely applied to bacterial communities. This is partially because it is often difficult to determine the pre-disturbance state of a population, definitions vary, and there is a lack of quantitative studies demonstrating how to implement these terms (*2*, *3*, *7*, *8*).

Yet, this resistance-resilience framework naturally lends itself to the analysis of bacterial responses to antibiotic treatment. When running susceptibility tests, it is possible to characterize a pre-disturbance state (i.e., no exposure to antibiotic) and there are many methods to quantify the bacterial antibiotic responses (i.e., agar plates, E-test, plate reader, and microscopy) (*9*–*11*). Until now, resistance and resilience have not been distinguished in the context of antibiotic responses. Instead, bacteria are classified as resistant if they survive exposure to a set concentration of antibiotic after a set amount of time (Table 1) (*12*). However, an apparently similar rate of survival can result from diverse underlying mechanisms (*13*, *14*): Survival can occur because individual cells withstand the treatment or because the population recovers from the initial disturbance, despite the fact that the antibiotic kills some individual bacterial cells. We term the former resistance and the latter resilience.

**Table 1:** Defining antibiotic responses. Each row represents a different response to an antibiotic delivered at time zero. In the first row, all individual cells can withstand the antibiotic, which results in the population growing to carrying capacity, unperturbed. In the second row, only a subpopulation of cells is killed. This result manifests as an initial decline in the population density followed by full recovery, after the antibiotic is removed in the allotted time. A third scenario entails a greater sensitivity compared with the second scenario, due to greater sensitivity of individual cells or lower capability of the population in removing the antibiotic. In this case, the population only partially recovers during the allotted time after the initial decline. In the final row, all cells are killed, leading to the population extinction. Currently, antibiotic sensitivity analyses only consider whether bacteria can recover from a set dose of antibiotic in a standard period (red dot). Bacteria that display full recovery are considered resistant, partial recovery are intermediate, and no recovery are sensitive. However, temporal dynamics (blue curve) reveal differences in how a population recovers. Living cells, blue; dead cells, gray. 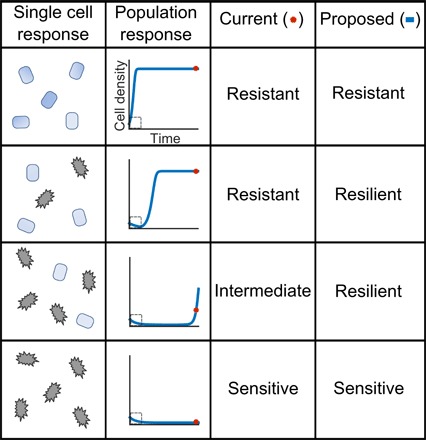

Here, we apply these concepts to the analysis of bacterial pathogens that produce extended-spectrum β-lactamases (ESBLs), which are becoming increasingly prevalent and can degrade many β-lactam antibiotics—the most widely used class of antibiotics (*15*). Our results offer new insights into the design of antibacterial treatment strategies against one of the most rapidly increasing types of bacterial pathogens (*16*–*18*). In particular, the resistance-resilience framework reveals the phenotypic signatures of different ESBL-producing bacteria and underscores the critical need to implement adjustable formulations of combination treatments. Our framework is also potentially applicable to analysis of bacterial population-level responses to other environmental perturbations, such as other antibiotics, changes in temperature and nutrients, and xeric stress.

## RESULTS

### Temporal dynamics of ESBL-producing bacteria in response to β-lactam treatment

**Fig. 1:**
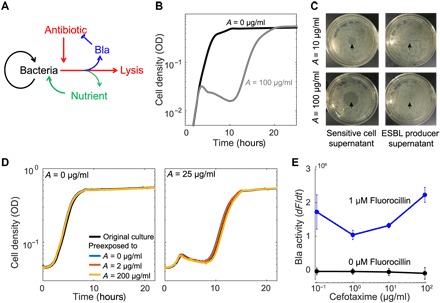
Response of an ESBL-producing population to cefotaxime, a β-lactam. (**A**) Schematic of antibiotic response of an ESBL-producing population. In the absence of antibiotic, bacteria reproduce and consume nutrients. Upon the introduction of an antibiotic, some of the bacteria undergo lysis and release Bla and a small amount of recyclable nutrients into the environment. The released Bla degrades the antibiotic (blue inhibition arm). (**B**) Time course of antibiotic response. In the absence of an antibiotic (black curve), bacteria grow to a carrying capacity without any delay. In the presence of sufficient antibiotic (gray curve; *A* = 100 μg/ml cefotaxime), the population displays the characteristic crash, as the cells lyse, and recovery after the Bla released from lysed cells degrades the antibiotic. (**C**) ESBL substantially degraded cefotaxime in a short time window. The supernatant from a culture of sensitive cells still contained substantial concentrations of cefotaxime, as depicted by the zones of inhibition in the lawn of sensitive cells (strain MC4100, left column). The supernatant from the culture containing ESBL-producing bacteria did not contain substantial concentrations of cefotaxime, as depicted by the full lawns (right column). Arrows indicate where supernatant was placed on the agar plate. (**D**) Populations previously exposed to cefotaxime exhibited the same temporal dynamics. Culture was treated with a range of antibiotic concentrations. After 24 hours, bacteria from the recovered population were used to reinoculate fresh media with or without cefotaxime (25 μg/ml). During the second round of treatment, time courses from the populations previously exposed to cefotaxime (0, 2, or 200 μg/ml) were identical, suggesting that the population recovery was unlikely due to mutants or phenotypic variants with increased tolerance. (**E**) Bla production is not induced by cefotaxime. We used fluorocillin to determine that the isolate’s Bla production is not significantly increased by the addition of antibiotic. Here, the Bla activity present in a population after 3 hours of exposure to a range of antibiotic concentrations was quantified by the rate at which fluorocillin was hydrolyzed and produced green fluorescence. One-way analyses of variance (ANOVAs) indicate that the increase in fluorescence recorded was insignificant when compared to the control.

The dynamics of an ESBL-producing population are uniquely suited for illustrating resistance and resilience during disturbances. In the absence of antibiotic treatment, the population grows approximately exponentially until the growth rate decreases because of the depletion of nutrients and accumulation of toxic compounds (Fig. 1A). Because of the expression of a β-lactamase (Bla) anchored in their periplasm, these bacteria can degrade the antibiotic that diffuses across the outer membrane (*19*). However, if Bla expression is moderate, these bacteria can still be lysed by a β-lactam antibiotic at a sufficiently high concentration (Fig. 1B). As this antibiotic effect occurs, Bla is released into the environment because of membrane leakage (from a cell not yet lysed) or cell lysis (*20*). If sufficient Bla (periplasmic and extracellular) is present, the antibiotic is degraded in time for the population to recover before all cells are lysed. A population’s recovery depends on collective tolerance (fig. S1): It must have a sufficiently high density when treated, so that enough Bla is collectively produced to remove the antibiotic before all bacteria are lysed. If the initial density is too low, then insufficient Bla will be produced to protect the population from antibiotic exposure.

Here, we showed that the population recovery was due to the Bla degrading the antibiotic, indicated by the level of antibiotic activity in the supernatant after 6 hours of exposure (Fig. 1C). Sensitive bacteria do not produce Bla and cannot break down the antibiotic; thus, the antibiotic remaining in the supernatant could inhibit the growth of sensitive bacteria. At a higher initial antibiotic concentration, the same amount of supernatant generated a larger zone of inhibition. In contrast, the bacteria producing ESBLs sufficiently degraded the antibiotic at both doses during the incubation period, as evidenced by the inability of the resulting supernatants to inhibit growth of sensitive cells. This Bla-dependent recovery was further demonstrated by the correlation between the time it takes for both sensitive and ESBL-producing bacteria to recover from antibiotic exposure and the amount of exogenous Bla present (fig. S2).

To test whether the population recovery was due to the selection of a more resistant or resilient subpopulation, we collected ESBL-producing bacteria that had recovered from an antibiotic treatment and reexposed them to a range of antibiotic concentrations. The resulting antibiotic responses were similar, regardless of previous exposure concentrations (Fig. 1D). This shows that the recovery was not due to the selection of a subpopulation with enhanced tolerance, which is consistent with the notion of antibiotic degradation due to the Bla released from cell lysis.

We also tested whether the antibiotic induced the production of Bla by using fluorocillin, a substrate that fluoresces green when degraded by Bla, allowing the real-time visualization of Bla activity. After incubating the isolate with different concentrations of cefotaxime for 3 hours, we added fluorocillin to the sonicated culture to quantify the total Bla present. There was no significant increase in fluorescence as a function of antibiotic concentration (Fig. 1E) at the *P* < 0.05 level (*F*_1,6_ = 2.44, *P* = 0.17 and *F*_1,6_ = 3.31, *P* = 0.12 for *A* = 10 and 100 μg/ml, respectively). There was a slight, but significant, decrease in fluorescence for *A* = 1 μg/ml (*F*_1,6_ = 6.68, *P* = 0.04). Overall, Bla production is not induced by exposure to this range of antibiotic concentrations.

### Defining resistance and resilience

**Fig. 2:**
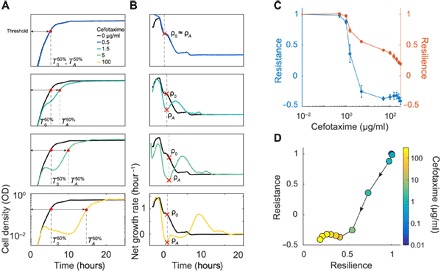
Quantifying resilience and resistance. (**A**) Time courses are used to quantify a population’s resilience. When no antibiotic was added (black curve), the population grew up unperturbed and reached a target threshold density (here, 50% of the carrying capacity) in time = *T*^50%^. If the antibiotic concentration added was very low (blue curve; *A* = 0.5 μg/ml cefotaxime), then the population reached the threshold density in a similar time to the untreated population. As the antibiotic concentration increased, the degree of lysis increased and affected the time necessary for the treated population to reach the threshold ($T_{A}^{50\%}$). We characterized the population’s resilience for a range of antibiotic concentrations as the inverse ratio of the times to the half-maximal carrying capacity ($T_{0}^{50\%}/T_{A}^{50\%}$). (**B**) Net growth rate quantifies population’s resistance. When no antibiotic was added, the population’s net growth rate decreased over time as it consumed the available nutrients and approached stationary phase. When a low dose of antibiotic was added, the net growth was not notably altered (blue curve). In this instance, the treated population’s minimum net growth rate is recorded as ρ_*A*_ and compared to the untreated population’s net growth rate at the same time (ρ_0_). As the antibiotic concentration increased, the net growth rate curve of the treated population deviated more from the untreated curve. For each antibiotic concentration, ρ_*A*_ was recorded as the net growth rate at the point of maximum negative deviation from the untreated population and normalized by ρ_0_. We characterized a population’s resistance as the ratio of recovery times (ρ_*A*_/ρ_0_). (**C**) Resistance and resilience as functions of the cefotaxime concentration. At low doses of cefotaxime, the population was resistant and resilient, showing little disturbance after exposure [see (A) and (B)]. As the antibiotic concentration was increased, resistance and resilience decreased due to the increase in cell lysis causing the net growth to decrease and the time to the half-maximum density increased. Once the population underwent a crash, the resistance was minimized and resilience became the dominating factor for survival. (**D**) The resistance-resilience map defines a phenotypic signature. Using the same data as in (A) to (C), the resistance-resilience framework can visualize the shift in a population’s antibiotic response. When the antibiotic concentration was 0 or very low, the population’s response displayed high resistance and resilience. Once the antibiotic concentration increased to 5 μg/ml, the population’s response shifted to a position where resistance was minimized and resilience dominated the antibiotic response. With further increase in antibiotic concentration, the resistance level continued to decrease. An effective treatment should minimize both resistance and resilience. Dot colors reference the antibiotic concentration used at that point, and arrows indicate the direction of increasing antibiotic concentrations.

A population can survive a disturbance because of its resistance or its resilience (*1*, *2*, *7*). In general, resistance refers to the ability of a population (or a community) to withstand the disturbance, whereas resilience refers to the ability to recover after suffering from the disturbance. Both properties are evident in the temporal dynamics of an ESBL-producing population in response to β-lactam treatment (Fig. 1). We quantify resilience as the rate of recovery by the population after experiencing the initial crash (Fig. 1A) by using the time needed for a population to reach 50% of its carrying capacity (*T*^50%^). With increasing antibiotic concentrations, more cells will lyse in the process of degrading the antibiotic, thus increasing the resulting recovery time. The more resilient a population is, the faster it can return to a normal state after being perturbed by an antibiotic. We define resilience as the inverse of the treated population’s *T*^50%^ ($T_{A}^{50\%}$), normalized by the untreated population’s *T*^50%^ ($T_{0}^{50\%}$) (Fig. 2A and fig. S3) (*1*). The inverse is taken to reflect the fact that increasing the recovery time corresponds to a decrease in resilience.

$$\text{Resilience}\;=\frac{T_{0}^{50\%}}{T_{A}^{50\%}}$$ (1)

By this definition, resilience reflects a long-term response and depends primarily on population-level traits: When a single bacterium can no longer survive the effects of antibiotic, the population is initially affected; however, collective antibiotic tolerance can allow the population to outlast the disturbance and recover after being perturbed.

We quantify resistance as the ability of the population to not deviate from the pre–antibiotic treatment state (as quantified by the growth rate). Mathematically, we define resistance as the ratio between the minimum net growth rate in the presence of an antibiotic in a treated population (ρ_*A*_) and the net growth rate of an untreated population (ρ_0_) at the same time. When dealing with the experimental data, our analysis accounts for the time delay in lysis caused by a β-lactam (*21*).

$$\text{Resistance}\;=\frac{\rho_{A}}{\rho_{0}}$$ (2)

By this definition, resistance primarily reflects the instantaneous response of individual cells but manifests at the population level. In particular, the magnitude and timing of measurement of the metric depends on the probability by which an average bacterium is lysed by the antibiotic (Fig. 2B). This probability is directly determined by the expression level and activity of Bla in the bacterium, as well as the extracellular concentration of the antibiotic. For a set amount of Bla, increasing the antibiotic concentration will require more time for the Bla to degrade the antibiotic, thus delaying the time at which minimum net growth rate is observed and resulting in more lysed bacteria and a smaller minimum net growth rate. In our analysis, we use optical density (OD) as a measure of the cell density.

For each bacterial strain, the degree of resistance or resilience depends on the type and dose of antibiotic used. At low antibiotic concentrations, the population experiences little or no disturbance and thus is characterized with relatively high resistance and resilience (Fig. 2C). At intermediate concentrations (*A* = 1.5 μg/ml), the population’s recovery displays a decline in both resistance and resilience because the antibiotic concentration is high enough to induce some lysis, slow the net growth rate, and delay the recovery time. Once the antibiotic concentration is high enough to induce a population crash (*A* > 5 μg/ml), the recovery of the population shifts to being dominated by resilience. If the antibiotic concentration exceeds the threshold for population recovery, the resulting resilience and resistance will be minimal. This resistance-resilience framework effectively reveals the phenotypic signature of each strain (Fig. 2D) when treated by a β-lactam.

In our experiments, the OD values are sufficiently small such that they are proportional to the total biomass (*21*). Debris from lysed cells can also contribute to the OD values. However, this contribution is negligible except for OD values that are near the baseline (when cells are lysed). Hence, the debris of lysed cells has negligible impact on calculated values of resistance or resilience.

### Determinants of resistance and resilience

**Fig. 3:**
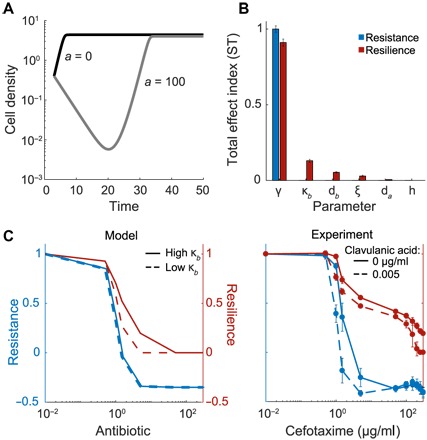
Modeling reveals key determinants of resistance and resilience. (**A**) Simulated time courses of an ESBL-producing isolate with and without an antibiotic. The characteristic “crash and recovery” is generated once the antibiotic concentration is high enough. (**B**) Sensitivity analysis reveals determinants of resistance and resilience. Total effect indices (ST) for resistance and resilience are reported for each parameter (*a* = 100 μg/ml). Resistance is most affected by the lysis rate (γ). The remaining parameters did not substantially affect the system’s resistance but did affect resilience. The most influential parameters included the maximum lysis rate (γ), Bla activity (κ_*b*_), the turnover rate of Bla (*d*_*b*_), and the amount of nutrients released during lysis (ξ). (**C**) Modulating resistance and resilience by tuning Bla activity. We altered Bla activity in the model (left column) or experimentally added clavulanic acid (right column) in combination with a range of antibiotic concentrations. Here, a low Bla activity corresponds to κ_*b*_ = 0 in the model or clavulanic acid (0.005 μg/ml) in the experiment. A high Bla activity corresponds to κ_*b*_ = 0. 35 in the model or no clavulanic acid in the experiment. Reducing Bla activity increased the population’s sensitivity, causing both resistance and resilience to decrease at lower concentrations of antibiotic.

To help further our investigation, we developed a kinetic model to describe the temporal response of a bacterial population constitutively expressing Bla to a β-lactam antibiotic. When no antibiotic is present, the population grows to carrying capacity without delay; however, once the antibiotic concentration is high enough, the population density undergoes a crash as a substantial portion of the population is lysed by the antibiotic, and a recovery as the released Bla degrades the antibiotic (Fig. 3A and fig. S4). We chose to simplify the system by lumping the activity of intra- and extracellular Bla, based on direct measurements that suggest that extracellular Bla plays a much greater role once substantial lysis has occurred (fig. S5).

Global sensitivity analysis was used to determine which parameters influenced resistance and resilience under a range of antibiotic concentrations. Briefly, the Sobol method calculates resilience and resistance for a range of parameter values and breaks down the variation for each into fractions that can be attributed to one or more parameters (*22*). Here, we reported the total effect index, ST, which reflects how much a parameter and all its interactions with any other parameters contributes to the variation in resistance and resilience at a particular antibiotic concentration (Fig. 3B and fig. S6). Comparing the ST for each parameter when *a* = 100 μg/ml reveals that resistance is only sensitive to the maximum lysis rate (ST_γ_ = 1 ± 0.02). The sensitivity analysis revealed that all parameters affect resilience to varying degrees, depending on the antibiotic concentration. Resilience is sensitive to the maximum lysis rate (ST_γ_= 0.9 ± 0.02), Bla activity ($ST_{\kappa_{b}}$= 0.13 ± 0.01), the turnover rate of Bla ($ST_{d_{b}}$= 0.05 ± 0.003), and the amount of nutrients recycled from cell lysis (ST_ξ_ = 0. 03 ± 0.01). These parameters determine the collective ability of the population to remove the antibiotic, underscoring the notion that resilience is a population-level trait.

We tested the influence of Bla activity computationally by varying κ_*b*_ and experimentally by using clavulanic acid, a well-characterized Bla inhibitor (Fig. 3C and fig. S7). With increased Bla inhibition, the population became more sensitive to the antibiotic, thus resulting in the antibiotic response shifting from relying on both resistance and resilience to just resilience at a lower concentration of antibiotic. Furthermore, the population with substantially reduced Bla activity did not survive exposure to the higher concentrations of antibiotic (resistance and resilience were both reduced).

### Phenotypic signatures of bacterial responses in the resistance-resilience framework

**Fig. 4:**
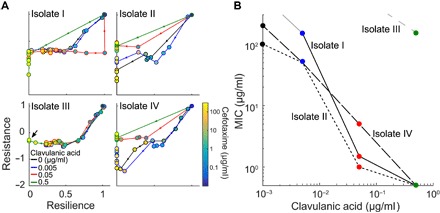
Diverse phenotypic responses by different ESBL-producing isolates. (**A**) Responses to combinations of Bla inhibitor and antibiotic concentrations. In general, with increasing antibiotic concentrations, the population responses start out as both resistant and resilient (top right of each subplot). The resistance is minimized with low doses of antibiotic, giving way to a response driven by resilience. As antibiotic concentration increased, the corresponding resilience decreased. With the addition of clavulanic acid, the concentration at which the response lost its resistance and then resilience is lowered. For isolates I, II, and IV, the highest clavulanic acid concentration (0.5 μg/ml, green curve) causes the antibiotic response to shift directly from resistant and resilient to sensitive with the lowest dose of cefotaxime (0.5 μg/ml). Isolate III was not as affected by clavulanic acid, requiring the highest concentration of the Bla inhibitor in combination with a high concentration of antibiotic to prevent the population from recovering (indicated by arrow). The color of the curves indicates the concentration of clavulanic acid. The color of the dots indicates the concentration of cefotaxime, and arrowheads indicate the direction of increasing antibiotic concentrations. (**B**) Dependence of MIC on Bla inhibition. For each ESBL-producing isolate, the MIC corresponds to the lowest concentration of cefotaxime needed to prevent recovery in 48 hours. One isolate, isolate III, was not very susceptible to clavulanic acid, suggesting that this intervention method would not be optimal in this case. Here, different line patterns represent the isolate, and the symbol color represents the concentration of clavulanic acid, as defined in (A). The gray lines indicate that the population recovered under all tested concentrations of cefotaxime tested at that concentration of clavulanic acid; therefore, the MIC could not be calculated (isolate I, clavulanic acid < 0.005 μg/ml; isolate IV, clavulanic acid < 0.5 μg/ml).

Given that Bla inhibitors are commonly used to restore sensitivity to some β-lactam antibiotics (*19*), we explored the implications of using the resistance-resilience framework to optimize combination treatments by analyzing the response of four different ESBL-producing isolates (table S1). These isolates were selected from a library obtained from the Durham Veterans Affairs Hospital, which included information regarding their species and whether they were expressing ESBLs. Two of the selected isolates (II and IV) had been characterized by whole-genome sequencing and were positive carriers for the *blaCTX-M-15* and *blaOXA-1* genes. These four isolates were chosen to demonstrate how the resistance-resilience framework could be applied without previous knowledge of the molecular mechanisms underlying the antibiotic response. We exposed each isolate to different combinations of antibiotic and Bla inhibitor concentrations and recorded their antibiotic responses. The resistance and resilience for each scenario were calculated and plotted against each other (Fig. 4A). The framework revealed how a small dose of antibiotic [cefotaxime (5 μg/ml)] could minimize resistance for isolate I, but larger doses were needed to minimize resilience. However, resilience could be minimized with a small dose of a Bla inhibitor [clavulanic acid (0.05 μg/ml)] when in combination with the antibiotic. A similar trend was observed in isolates II and IV, with treatments using higher concentrations of the Bla inhibitor requiring less antibiotic to minimize resistance and resilience. Isolate III, however, was not affected by increasing concentrations of the Bla inhibitor, as seen by the overlapping resistance-resilience curves. Only the highest concentration of the Bla inhibitor (0.5 μg/ml) in combination with a high concentration of antibiotic (150 μg/ml) prevented the population from recovering. In this study, resistance and resilience are coupled for the different clinical isolates. This is, in part, due to the expression of Bla that mediates resistance for single cells and the resilience for the population. In general, however, as Nimmo *et al*. explained, resistance and resilience are not always interdependent (*2*).

Using the resistance-resilience framework, we determined that the minimum inhibitory concentration (MIC) of antibiotic for each level of the Bla inhibitor was unique for each isolate (Fig. 4B). Here, the MIC is defined as the concentration necessary to prevent the population from recovering within 48 hours of being exposed to treatment. For example, when clavulanic acid (0.05 μg/ml) was used, MIC values of cefotaxime were 5, 1.5, >300, and 1 μg/ml for isolates I, II, III, and IV, respectively. This diversity in the treatment responses can be explained by the expression of different or additional types of Bla that exhibit different sensitivities to inhibition by clavulanic acid. For instance, isolate III’s lack in response is likely due to the production of cephalosporinases or chromosomally mediated Bla, which have been shown to be poorly inhibited by clavulanic acid (*19*, *23*). Isolate IV is less sensitive to the initial increases of clavulanic acid; thus, it needs higher concentrations of cefotaxime, relative to isolates I and II, to prevent the population from regrowing at the second highest level of Bla inhibition. These results underscore a critical caveat in using predetermined formulations of β-lactam/Bla inhibitor combinations to combat ESBL-producing pathogens, which is currently a standard practice (*19*, *24*, *25*). Given the diversity of the phenotypic responses by the different isolates, quantitative measurements on how a strain responds to an antibiotic and a Bla inhibitor are necessary to predict the outcome of a particular combination treatment.

## DISCUSSION

Resistance and resilience provide a powerful framework to dissect the contributions of different factors underlying a population’s response to a disturbance. Despite their appeal, resistance and resilience have had limited applications for understanding population responses due to differing definitions (*1*, *2*) and a lack of quantitative studies. Our analysis of an ESBL-producing clinical isolate’s response to a β-lactam antibiotic serves as a concrete example of the dichotomy between resistance associated with single cells and resilience associated with populations. That is, resilience can be considered a cooperative trait by a group of bacteria (clonal or mixed).

Our work provides a concrete procedure to quantify resistance and resilience in a population (clonal or mixed) in response to neutral or negative perturbations, as both metrics can be uniquely defined from the time course of the population, as long as the minimum net growth rate of the treated population can be measured before the control population reaches its carrying capacity (e.g., before ρ_0_ = 0). Hence, it can be applied to diverse situations. For example, some bacteria are resistant to xeric stress due to the disturbance triggering their adaptive mechanisms (*26*). Specifically, a xerotolerant cell survives a dry spell by decreasing its energy consumption, protecting its DNA from damaging reactive oxygen species, stabilizing its membrane, and preventing intracellular water loss. Another example of resistance being a single-cell level response is the production of heat shock proteins to enable the cell’s survival of stressful conditions, such as extreme temperatures (*27*). As for resilience, a population of cyanobacteria has been shown to depend on its density to survive high levels of light that are damaging to single cells. Mutual shading is a density-dependent phenomenon achieved when the damaged cyanobacteria that are closer to the light source provide shade to their lower neighbors, thus allowing the population to regrow in lower, less damaging levels of light (*28*). Bacterial resilience to antimicrobial peptides can also be conferred by outer membrane vesicles (OMVs) (*29*). Treatment by an antimicrobial peptide (at a high enough concentration) leads to lysis of a subpopulation of cells, leading to generation of OMVs. The OMVs can adsorb the antimicrobial peptide and allow eventual population recovery. In addition, the microbiome is resilient to diet changes, antibiotic exposure, and invasion by new species due to population-level attributes such as species richness and functional redundancy afforded through species diversity (*30*).

In our analysis, the framework is applied to the dynamics of a homogeneous population, where resistance and resilience both depend on the average properties of cells. However, this framework is also applicable for dissecting bacterial responses to antibiotics for heterogeneous populations (fig. S7). For instance, cell-to-cell variability in gene expression can cause fluctuations in single-cell growth and death rates when in the presence of a disturbance (*31*, *32*). Here, this could manifest as a distribution of subpopulations expressing different levels of intracellular ESBLs that convey different costs (i.e., reducing growth rate) and benefits (i.e., offering protection from antibiotic) to the subpopulations in the presence of an antibiotic (*33*). In the extreme case, a population’s antibiotic response can be driven by a small subpopulation of persisters or slow-growing or dormant bacteria that are not sensitive to antibiotics (*11*). Upon antibiotic exposure, most of the population is killed, leaving the persisters behind. Once the antibiotic has been removed, the persisters, which are genetically identical to their sensitive counterparts, spontaneously switch back into the normal, growing state and reestablish the colony. This antibiotic response would be characterized with low resistance and high resilience due to the presence of persisters.

Alternatively, bacteria that have mutated forms of a given antibiotic’s target are resistant to that antibiotic (*34*). For instance, vancomycin-resistant *Enterococcus faecalis* (VRE) has mutated the end of a peptidoglycan strand, a component necessary for cell wall synthesis and the target for vancomycin (*35*). This mutation reduces the peptidoglycan’s binding affinity for vancomycin by 1000-fold. If a single VRE cell has this mutation and thus the ability to withstand much higher antibiotic concentrations, an entire population’s antibiotic response would be characterized as high resistance.

Our framework is connected to another in quantifying bacterial responses. In particular, Artemova and colleagues (*36*) introduced the concept of single-cell MIC (scMIC) to describe the susceptibility of individual bacteria to a β-lactam antibiotic. This concept is in contrast to the concept of MIC, which is typically determined as the collective response of a population. The scMIC concept operates at the same level as resistance described in our study. Both scMIC and resistance reflect the ability of individual cells to survive an antibiotic except that resistance reflects the antibiotic resistance of an average cell. Thus, both scMIC and resistance depend on parameters associated with individual cells, including the maximum lysis rate. In contrast, resilience and MIC result from the collective behavior of the entire population. Both also depend on the inoculum size of the population, but resilience has the advantage of accounting for the temporal dynamics.

In addition to dissecting an antibiotic response into its components and corresponding attributes, the resistance-resilience framework will be a useful tool for investigators to improve the design of combination treatments. Comparing the resistance-resilience fingerprint of different isolates under varied concentrations of cefotaxime and clavulanic acid revealed how varied their responses are to Bla inhibition. As one way to extend the efficacy of a β-lactam is by pairing it with a Bla inhibitor (*37*), this observation is relevant for guiding the optimization of combination treatments. Currently, there are a few versions of the treatment combining clavulanic acid and amoxicillin for clinical use; however, the concentration of clavulanic acid is kept constant between the versions while the amoxicillin concentration is changed (*38*, *39*). Early studies suggest that this clavulanic acid concentration was selected to minimize patient side effects and maintain a sufficiently high serum concentration of clavulanic acid (*19*, *24*, *25*). Nevertheless, our finding suggests that the clavulanic acid concentration can be optimized within a safe range to reduce the amount of antibiotic necessary and minimize the resistance and resilience of a given isolate. Furthermore, a recent study found that different ratios of inhibitor to antibiotic could influence the rate and mechanism of antibiotic resistance that bacteria develop (*40*).

## METHODS

### Bacterial strains, growth media, and culturing conditions

We characterized bacterial isolates from a library assembled by the Duke Hospital’s Division of Infectious Diseases. This library consists of approximately 80 isolates that have been identified as ESBL producers. Unless otherwise noted, *Klebsiella pneumoniae* isolate DICON 005 was used. As a sensitive bacteria control, *Escherichia coli* MC4100 cells were used. Unless otherwise indicated, experiments were conducted in M9 medium [1× M9 salts (48 mM Na_2_HPO_4_, 22 mM KH_2_PO_4_, 862 mM NaCl, and 19 mM NH_4_Cl), 0.4% glucose, 0.2% casamino acids (Teknova), 0.5% thiamine (Sigma), 2 mM MgSO_4_, and 0.1 mM CaCl_2_]. For overnight cultures, we inoculated single colonies from an agar plate into 2 ml of M9 and incubated them for 12 hours at 30°C.

### Measuring time courses

One milliliter of overnight culture was washed (centrifuged for 5 min at 13,000 rpm, discarded the supernatant, and resuspended in 1 ml of fresh M9), and the OD was adjusted to 0.5 OD_600_ by adding the appropriate volume of M9 (fig. S9). The culture was then diluted 1000-fold in fresh M9, and the appropriate amount of cefotaxime (Sigma) was added to achieve a range of concentrations from 0 to 300 μg/ml. A 96-well plate (Costar) was loaded with 200 μl of culture per well and topped with 50 μl of mineral oil (Sigma) to prevent evaporation. The plate was loaded into a Tecan Infinite M200 PRO microplate reader (chamber temperature maintained at 30°C), and OD_600_ readings were measured every 10 min for 48 hours with intermittent plate shaking. Unless otherwise noted, each condition tested consisted of four technical replicates that, when averaged, did not need to include error bars.

### Cefotaxime activity level

ESBL-producing isolate I and sensitive strain MC4100 were cultured in M9 for 12 hours at 30°C (fig. S10). One milliliter of each overnight culture was washed (centrifuged for 5 min at 13,000 rpm, discarded the supernatant, and resuspended in 1 ml fresh M9), and the OD was adjusted to 0.5 OD_600_ by adding the appropriate volume of M9. The culture was then diluted 1000-fold in 4 ml of fresh M9, and the appropriate amount of cefotaxime was added to achieve final concentrations of 0, 10, or 100 μg/ml. The cultures were incubated at 37°C for 6 hours. At this time, lawns of sensitive cells were prepared by spreading 5 μl of the 1000-fold diluted MC4100 culture onto agar plates. Supernatant from the cultures incubated with cefotaxime were prepared by spinning down 0.5 ml of culture with clavulanic acid (5 μg/ml; to prevent further Bla activity). Four microliters of the supernatant was dropped into the center of the agar plates, which were then incubated for 16 hours at 37°C. The zones of inhibition were recorded by a camera.

### Varying exogenous Bla

Overnight cultures of ESBL isolates I to IV and sensitive cells (MG1655) were prepared by inoculating a colony in LB for 12 hours at 37°C. ESBL cultures were washed and diluted, as in the “Measuring time courses” section, and the appropriate amount of cefotaxime (Sigma) was added to achieve 50 μg/ml in M9. Exogenous Bla (Sigma) was prepared in phosphate-buffered saline (PBS) and added to the ESBL cultures to achieve concentrations ranging from 1 to 100 μg/ml. MG1655 was washed and diluted, as in the “Measuring time courses” section, and the appropriate amount of carbenicillin (Genesee) was added to achieve 100 μg/ml. Exogenous Bla was prepared in PBS and added to the MG1655 cultures to achieve concentrations ranging from 1 to 20 μg/ml. Time courses were measured by a Tecan Spark and a Tecan M200 plate reader for the ESBL isolates and MG1655, respectively, as in the “Measuring time courses” section.

### Selective pressure

After conducting a 24-hour time course, as described in the “Measuring time courses” section, cultures that had been exposed to cefotaxime doses of 0, 2, and 200 μg/ml were diluted 10-fold in fresh M9 (antibiotic free) and incubated at 37°C for 3 hours (fig. S11). The recovered cultures were then used to run another time course using the same antibiotic concentrations used in the previous round of treatment.

### Quantifying Bla activity

One milliliter of overnight culture was washed (centrifuged for 5 min at 13,000 rpm, discarded the supernatant, and resuspended in 1 ml fresh M9) and diluted 1000-fold in fresh M9. The appropriate amount of cefotaxime (Sigma) was added to achieve 0, 1, 10, and 100 μg/ml. The cultures were incubated for 3 hours at 30°C. For each culture, 1 ml was kept on ice to preserve the population density at the 3-hour time point. Two milliliters was spun down (5 min, 13,000 rpm) and resuspended in 2 ml of water before being sonicated (20 amp, duration of 1 min at 4°C) to release periplasmic Bla. The sonicated culture was diluted 10-fold in water and then treated with 0 or 1 μM fluorocillin. A 96-well plate was loaded with 200 μl of each culture (whole cells), sonicated with and without fluorocillin, and topped with 50 μl of mineral oil. The plate was loaded into a Tecan Infinite M200 PRO microplate reader (chamber temperature maintained at 25°C), and OD_600_ and green fluorescent protein (GFP) readings were measured every 10 min for 1.5 hours. The GFP measurements of the sonicated samples were plotted over time, and the slope was calculated. The slope was normalized by the relevant culture’s OD measurement. A one-way ANOVA was used to determine any significant differences between the conditions.

### Quantifying internal versus external Bla activity

ESBL-producing isolates were incubated with cefotaxime (0, 0.01, 1, 10, and 100 μg/ml) for 12 hours at 30°C. At that point, the culture was centrifuged (13,000 rpm, 5 min) to separate the supernatant and whole cells. The supernatant was removed, and whole cells were resuspended in fresh M9. A portion of the whole cells was removed and sonicated (20 amp, duration of 1 min at 4°C) to release Bla from the periplasmic space. Bla activity in each component (supernatant, sonicated whole cells, and whole cells) was quantified by adding 1 μM fluorocillin and monitoring the change in green fluorescence with a plate reader. A 96-well plate was loaded with 200 μl of each mixture and topped with 50 μl of mineral oil. The plate was loaded into a Tecan Infinite M200 PRO microplate reader (chamber temperature maintained at 25°C), and OD_600_ and GFP readings were measured every 10 min for 1.5 hours. The GFP measurements of the samples were plotted over time, and the slope was calculated. The slope was normalized by the whole cell’s OD measurement.

### Varying Bla inhibition

The preparation of cells, antibiotic, and 96-well plate were prepared as in the “Measuring time courses” section. When the cefotaxime was added, clavulanic acid potassium salt (Sigma) was also added to achieve final concentrations ranging from 0 to 5 μg/ml.

### Varying initial cell density

Culture was prepared as in the “Bacterial strains, growth media, and culturing conditions” section except that the culture dilution ranged from 100- to 100,000-fold. Cultures were exposed to cefotaxime (0 or 100 μg/ml), time courses were measured by a plate reader, and the final cell density after 30 hours was recorded.

### Plate reader data analysis

MATLAB (version 9.2.0.556344, R2017a) was used to plot and characterize the time courses obtained from the plate reader (i.e., recovery time, growth rate, and change in GFP over time).

### Modeling

The interaction between a β-lactam and a bacterial population expressing a Bla can be simplified to the interactions between four main components: population density, antibiotic concentration, nutrient level, and Bla concentration. To model bacteria that constitutively produce Bla and lyse due to antibiotics degrading the cell wall, we modified Tanouchi *et al*.’s ordinary differential equation model (*41*) for the nondimensional dynamics of bacterial density (*n*), extracellular Bla concentration (*b*), nutrient level (*s*), and β-lactam concentration (*a*).

$$\frac{dn}{d\tau }=(g-l)n$$ (3)

$$\frac{db}{d\tau }=ln-d_{b}b$$ (4)

$$\frac{da}{d\tau }=-\kappa_{b}ba-d_{a}a$$ (5)

$$\frac{ds}{d\tau }=(\xi l-g)n$$ (6)

$$g=\frac{s}{1+s}$$ (7)

$$l=\gamma \frac{a^{h}}{1+a^{h}}g$$ (8)

Initial conditions of *n*(0) = 0.4, *b*(0) = 0, *a*(0) = 0:300, and *s*(0) = 4 were used for all the simulations. We assume that the growth rate of cells (*g*) is limited by *s*, following the Monod kinetics. We assume the lysis rate (*l*) can reach a maximum of γ and depends on *a* following the Hill kinetics and *g*. *d*_*B*_ and *d*_*A*_ are the natural decay rates of extracellular Bla and antibiotic, respectively. κ_*b*_ is the rate at which Bla degrades the antibiotic. ξ is a weighting factor for how much nutrients are recycled upon cell lysis, and *h* is the Hill coefficient. See the Supplementary Materials for parameter values and full model development.

## Supplementary Material

http://advances.sciencemag.org/cgi/content/full/4/12/eaau1873/DC1

## SUPPLEMENTARY MATERIALS

Supplementary material for this article is available at http://advances.sciencemag.org/cgi/content/full/4/12/eaau1873/DC1 Section S1. Model development Section S2. Sensitivity analysis Fig. S1. Collective antibiotic tolerance. Fig. S2. Varying exogenous Bla. Fig. S3. Time course and rate of change curves of isolate I. Fig. S4. Time courses and rate of change curves generated by the model. Fig. S5. Quantifying Bla activity in different components of culture. Fig. S6. Sensitivity analysis reveals parameters affecting resistance and resilience. Fig. S7. Time courses showing the effects of Bla inhibition. Fig. S8. Framework can be applied to heterogeneous populations. Fig. S9. Schematic for methods. Table S1. ESBL-producing isolates screened. References (*42*–*52*)
