# Supplementary material for: Applying ecological resistance and resilience to dissect bacterial antibiotic responses
Source: Sci Adv. 2018 Dec 5;4(12):eaau1873. doi: 10.1126/sciadv.aau1873 (PMC6281428; doi:10.1126/sciadv.aau1873)
Supplement: http://advances.sciencemag.org/cgi/content/full/4/12/eaau1873/DC1 [file supp_4_12_eaau1873__index.html]

Science Advances | Science Advances

## Supplementary Materials

**This PDF file includes:**

- Section S1. Model development
- Section S2. Sensitivity analysis
- Fig. S1. Collective antibiotic tolerance.
- Fig. S2. Varying exogenous Bla.
- Fig. S3. Time course and rate of change curves of isolate I.
- Fig. S4. Time courses and rate of change curves generated by the model.
- Fig. S5. Quantifying Bla activity in different components of culture.
- Fig. S6. Sensitivity analysis reveals parameters affecting resistance and resilience.
- Fig. S7. Time courses showing the effects of Bla inhibition.
- Fig. S8. Framework can be applied to heterogeneous populations.
- Fig. S9. Schematic for methods.
- Table S1. ESBL-producing isolates screened.
- References (*42*–*52*)

Download PDF

**Files in this Data Supplement:**

- Adobe PDF - aau1873\_SM.pdf
