# Supplementary material for: Applying ecological resistance and resilience to dissect bacterial antibiotic responses
Source: Sci Adv. 2018 Dec 5;4(12):eaau1873. doi: 10.1126/sciadv.aau1873 (PMC6281428; doi:10.1126/sciadv.aau1873)
Supplement: http://advances.sciencemag.org/cgi/content/full/4/12/eaau1873/DC1 [file aau1873_SM.pdf]

Science Advances
AAAS

advances.sciencemag.org/cgi/content/full/4/12/eaau1873/DC1

Supplementary Materials for

Applying ecological resistance and resilience to dissect bacterial antibiotic responses

Hannah R. Meredith, Virgile Andreani, Helena R. Ma, Allison J. Lopatkin, Anna J. Lee, Deverick J. Anderson, Gregory Batt, Lingchong You*

*Corresponding author. Email: you@duke.edu

Published 5 December 2018, *Sci. Adv.* **4**, eaau1873 (2018)
DOI: 10.1126/sciadv.aau1873

This PDF file includes:

Section S1. Model development
Section S2. Sensitivity analysis
Fig. S1. Collective antibiotic tolerance.
Fig. S2. Varying exogenous Bla.
Fig. S3. Time course and rate of change curves of isolate I.
Fig. S4. Time courses and rate of change curves generated by the model.
Fig. S5. Quantifying Bla activity in different components of culture.
Fig. S6. Sensitivity analysis reveals parameters affecting resistance and resilience.
Fig. S7. Time courses showing the effects of Bla inhibition.
Fig. S8. Framework can be applied to heterogeneous populations.
Fig. S9. Schematic for methods.
Table S1. ESBL-producing isolates screened.
References (*42–52*)

Section S1. Model development

We model the dynamics of a system of 4 variables: the cell density (N), the nutrient concentration (S), the antibiotic concentration (A) and the external Bla concentration (B).

Reactions

$$\mathrm{N} + \mathrm{S} \xrightarrow{\text{Monod}} 2\,\mathrm{N} \qquad \mathrm{N} \xrightarrow{L(A,S)} B_{\text{in}}\,\mathrm{B} + \xi\mathrm{S} \tag{1}$$

$$\mathrm{A} \xrightarrow{d_{\mathrm{A}}} \varnothing \qquad \mathrm{B} \xrightarrow{d_{\mathrm{B}}} \varnothing \tag{2}$$

$$\mathrm{B} + \mathrm{A} \underset{k_2}{\overset{k_1}{\rightleftharpoons}} \mathrm{BA} \qquad \mathrm{BA} \xrightarrow{k_3} \mathrm{B} \tag{3}$$

1. Left: A cell, plus an equal mass of nutrients, produces another cell with a Monod dynamics. Right: Cells lyse at a rate L (which depends on A and S), releasing a quantity B_{in} of Bla and a proportion of nutrients ξ.
2. The antibiotic and Bla decay respectively at rates d_{A} and d_{B}.
3. Enzymatic degradation of the antibiotic by Bla (outside the cells).

Units In order to derive differential equations straightforwardly from this reaction network, adequate units need to be chosen for the state variables and parameters. We will count N and S in the same unit, number of (equivalent) bacteria per litre, and A and B in moles per litre. In the following, as a visual aid, we will note $|X|$ all quantities in the unit of N, and $[X]$ all quantities in the unit of A

$$\begin{aligned}
\frac{\mathrm{d}|N|}{\mathrm{d}t} &= G|N| - L|N| & G &= \mu\frac{|S|}{|K_S| + |S|} \\
\frac{\mathrm{d}|S|}{\mathrm{d}t} &= -G|N| + \xi L|N| & L &= \gamma\frac{[A]^h}{[K_A]^h + [A]^h}G \\
\frac{\mathrm{d}[A]}{\mathrm{d}t} &= -k_1[B][A] + k_2[BA] - d_{\mathrm{A}}[A] \\
\frac{\mathrm{d}[B]}{\mathrm{d}t} &= B_{\text{in}}L|N| - d_{\mathrm{B}}[B] - k_1[B][A] \\
&\quad + k_2[BA] + k_3[BA] \\
\frac{\mathrm{d}[BA]}{\mathrm{d}t} &= k_1[B][A] - k_2[BA] - k_3[BA]
\end{aligned}$$

A quasi-steady-state approximation on the transitory complex BA yields $[BA] = \frac{k_1}{k_2+k_3}[B][A]$. Replacing this expression into the system gives

$$\frac{\mathrm{d}|N|}{\mathrm{d}t} = (G - L)|N| \qquad G = \mu \frac{|S|}{|K_S| + |S|}$$

$$\frac{\mathrm{d}|S|}{\mathrm{d}t} = (\xi L - G)|N| \qquad L = \gamma \frac{[A]^h}{[K_A]^h + [A]^h} G$$

$$\frac{\mathrm{d}[A]}{\mathrm{d}t} = -k_b[B][A] - d_\mathrm{A}[A] \qquad k_b = \frac{k_1 k_3}{k_2 + k_3}$$

$$\frac{\mathrm{d}[B]}{\mathrm{d}t} = B_\mathrm{in} L|N| - d_\mathrm{B}[B]$$

Dimensionless model In order to make the model more suitable for simulations, and factor out some unidentifiable parameters, we made it dimensionless by the following change of variables

$$n = \frac{|N|}{|K_S|} \qquad s = \frac{|S|}{|K_S|}$$

$$a = \frac{[A]}{[K_A]} \qquad b = \frac{[B]}{B_\mathrm{in}|K_S|}$$

$$\kappa_b = \frac{k_b B_\mathrm{in}|K_S|}{\mu} \qquad \tau = t\mu$$

$$d_a = \frac{d_A}{\mu} \qquad d_b = \frac{d_B}{\mu}$$

Resulting in this dimensionless model

$$\frac{\mathrm{d}n}{\mathrm{d}\tau} = (g - l)\, n \qquad g = \frac{s}{1+s}$$

$$\frac{\mathrm{d}s}{\mathrm{d}\tau} = (\xi l - g)\, n \qquad l = \gamma \frac{a^h}{1 + a^h}\, g$$

$$\frac{\mathrm{d}a}{\mathrm{d}\tau} = -\kappa_b\, b\, a - d_a\, a$$

$$\frac{\mathrm{d}b}{\mathrm{d}\tau} = l\, n - d_b\, b$$

Heterogeneous model Heterogeneity was introduced into the model by dividing the population into subpopulations (i) with unique growth rates selected from a normal distribution of values (π) and equal starting densities

$$\frac{\mathrm{d}n_i}{\mathrm{d}\tau} = (g_i - l_i)\, n_i \qquad g_i = \pi_i \frac{s}{1+s}$$
$$\frac{\mathrm{d}s}{\mathrm{d}\tau} = \sum_{i=1}^{n} (\xi l_i - g_i)\, n_i \qquad l_i = \gamma \frac{a^h}{1+a^h}\, g_i$$
$$\frac{\mathrm{d}a}{\mathrm{d}\tau} = -\kappa_b\, b\, a - d_a\, a$$
$$\frac{\mathrm{d}b}{\mathrm{d}\tau} = \sum_{i=1}^{n} l_i\, n_i - d_b\, b$$

Parameter values and typical ranges

Initial values In all the experiments, a 3-hour delay is observed before the crash becomes visible (see S. Fig. 3 for example). We didn't model this delay, but assumed that the growth was nominal during this initial phase, and started all the simulations from the state reached after 3 hours of normal exponential growth. Because of collective antibiotic tolerance, to conserve a potent antibiotic on a higher cell density, we had to underestimate the efficiency of the antibiotic hydrolysis by Bla (κ_b). This is indeed the parameter that deviates the most from the literature values.

Dimensioned model				Dimensionless model		
Variable	Typical range	Initial value	Unit	Variable	Typical range	Initial value
$\lvert N\rvert$	10^3 to 10^{12}	$7 \cdot 10^{10}$	cell/L	$n = \frac{\lvert N\rvert}{\lvert K_S\rvert}$	10^{-8} to 10	0.4
$\lvert S\rvert$	10^3 to 10^{12}	$4 \cdot 10^{12}$	cell/L	$s = \frac{\lvert S\rvert}{\lvert K_S\rvert}$	10^{-8} to 10	4
$[A]$	0 to 10^{-3}	0 to 10^{-3}	mol/L	$a = \frac{[A]}{[K_A]}$	0 to 500	0 to 500
$[B]$	0 to 10^{-7}	0	mol/L	$b = \frac{[B]}{B_{\mathrm{in}}[K_S]}$	0 to 3	0

Analytical expression of resistance It is possible to derive from this model an analytical expression for the resistance. Indeed, the resistance is defined as $\frac{\rho_A}{\rho_0}$, where ρ_A is the maximum net lysis rate observed, and ρ_0 is the maximum growth rate observed without antibiotic. The maximum lysis rate is always observed at the introduction of the antibiotic (after the 3-hour delay), when the antibiotic concentration in the medium is still unaltered. The net lysis rate is then $\rho_A = G - L = G - \gamma\frac{[A]^h}{[K_A]^h+[A]^h}G$, and taking $\rho_0 = G$ we finally obtain

$$\text{Resistance} = 1 - \gamma\frac{[A]^h}{[K_A]^h + [A]^h} = 1 - \gamma\frac{a^h}{1+a^h}$$

Dimensioned model				Dimensionless model	
Param.	Literature value	Unit	Ref.	Parameter	Fitted value
μ	0.8	1/h	[41]	-	-
$\lvert K_S \rvert$	$4 \cdot 10^9$ to 10^{11}	cell/L	[48]	-	-
ξ		1	[46]	ξ	0.8
γ	1.8 to 4	1	[49]	γ	1.35
$[K_A]$	$2 \cdot 10^{-6}$	mol/L	[45]	-	-
h	3	1	[44]	h	3
k_b	10^{10}	$\mathrm{L/(mol \cdot h)}$	[47]	$\kappa_b = k_b \frac{B_{\mathrm{in}} \lvert K_S \rvert}{\mu}$	0.35
d_{A}	0.015	1/h	[42]	$d_a = \frac{d_{\mathrm{A}}}{\mu}$	0.02
d_{B}	0.02 to 0.5	1/h	[52]	$d_b = \frac{d_{\mathrm{B}}}{\mu}$	0.1
B_{in}	$3 \cdot 10^{-19}$	mol/cell	[50]	-	-
D	$1 \cdot 10^{-12}$	OD/(cell/L)	[51]	-	-

A simple analytical expression does not seem to exist for the resilience.

Section S2. Sensitivity analysis

Sobol sensitivity analysis was used to determine which parameters influenced resistance and resilience under a range of antibiotic concentrations (see the tutorial by Zhang [22] for a good explanation). Briefly, the total-order sensitivity index, ST, reflects how much a parameter contributes to the variation of resistance or resilience, alone or in coordination with any number of others. The sensitivity analysis was performed on the dimensionless model, on a parameter space spanning a factor of 4 centered around each parameter's default value ($x \in [\frac{x_0}{2}, 2x_0]$). The sampling of the parameter space and analysis of the results was done with the open-source Python library SALib[43].

2.1 Resistance

The main effector of resistance is the maximum lysis rate γ, which is given a total-effect index of 1 at 1, 10 and 100 µg/mL.

From the expression derived earlier

$$\text{Resistance} = 1 - \gamma \frac{a^h}{1 + a^h}$$

When $a \ll 1$, resistance will be 1, regardless of parameter variation. When $a \gg 1$, resistance becomes dependent on γ as resistance approaches $1 - \gamma$. When $a \approx 1$, h should have a minor influence, which is shadowed by γ's. These parameters are characteristic of single cell level behavior, suggesting that resistance is a single cell level trait.

2.2 Resilience

Results from the sensitivity analysis for resilience revealed that all parameters contributed to varying degrees, depending on the antibiotic concentration. At low concentrations of antibiotic, the most influential parameters are the maximum lysis rate ($ST_\gamma = 0.81 \pm 0.02$) and the catalytic activity of Bla ($ST_{\kappa_b} = 0.145 \pm 0.005$). At higher concentrations, γ and κ_b's total-effect indexes persist as the two mainly influential parameters, with the addition of the decay rate of Bla which appears at $ST_{d_b} = 0.040 \pm 0.002$ at $A = 10\,\mu g/mL$ and at $ST_{d_b} = 0.053 \pm 0.003$ at $A = 100\,\mu g/mL$. Although γ is the main effector, κ_b and d_b are related to population-level behaviors, suggesting that resilience could be a population-level trait.

Supplementary figures and tables

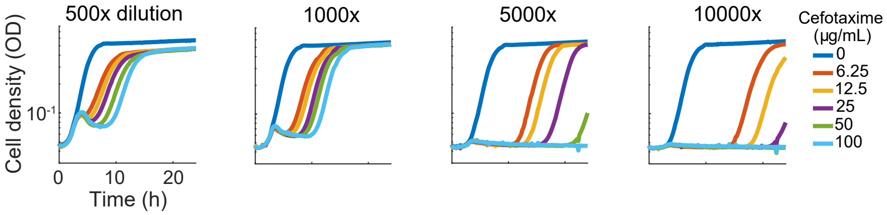

Fig. S1. Collective antibiotic tolerance. The population survival to antibiotic exposure depends on the initial cell density. The starting density was diluted from 500 to 10000 times and then exposed to a range of cefotaxime doses. The time courses show that increasing the antibiotic concentration resulted in a longer recovery time because more cells were lysed. If the cell density is too low, then there will be too few cells to produce the amount of Bla needed to degrade the antibiotic and allow the population to recover.

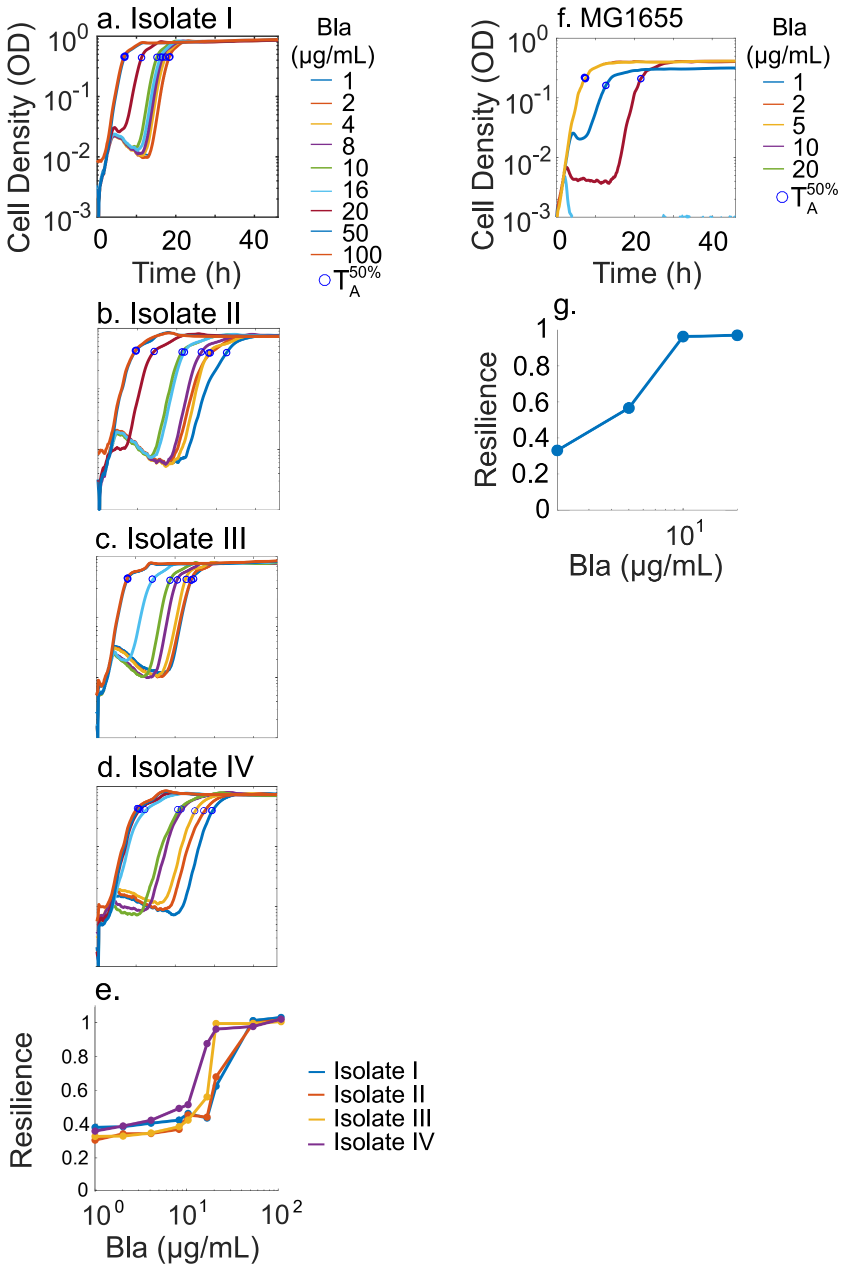

Fig. S2. **Varying exogenous Bla.** Time courses and resistance and resilience plots showing the effects of exogenous Bla addition on the antibiotic response of ESBL-producing and sensitive populations.(a-d) Four ESBL-producing isolates were exposed to 50 µg/mL cefotaxime in combination with exogenous Bla ranging from 1 to 100 µg/mL. Increasing Bla concentrations reduced the recovery time and increased resilience (e) for all isolates in a dose-dependent fashion. (f) Sensitive (MG1655) cells were exposed to 100 µg/mL of carbenicillin in combination with exogenous Bla ranging from 1 to 20 µg/mL. Increasing Bla concentrations reduced the recovery time resilience (g).

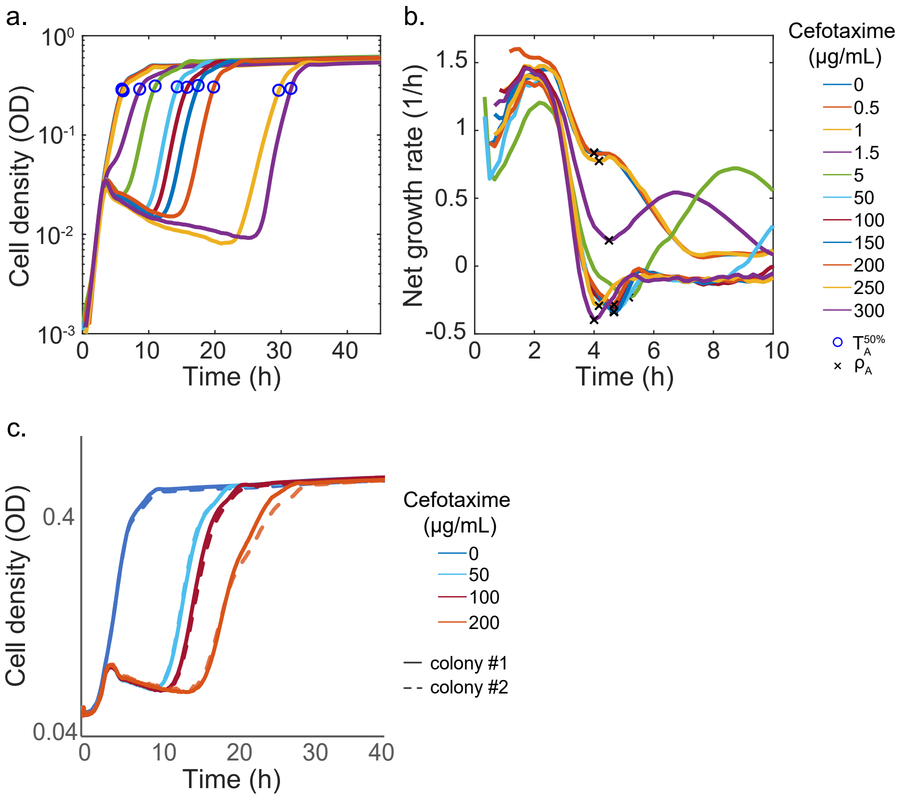

Fig. S3. Time course and rate of change curves of isolate I. (a) Time courses were measured for a range of antibiotic concentrations (added at time = 0). At low concentrations ($A < 1.5\,\mu g/mL$), the growth curves looked very similar to the control ($A = 0$) and are considered to be "resistant" to these conditions. At $A = 1.5\,\mu g/mL$, the growth curve starts to deviate from the control due to an increase in cell lysis, showing a decrease in resistance. Once the concentration was high enough ($A \geq 5\,\mu g/mL$), the population started displaying the crash and recovery dynamics. The time to half maximum density ($T^{50\%}$, blue circle) was determined for each concentration and used to calculate resilience. (b) Resistance was calculated from the net growth rate. ρ_A is calculated as the point of maximum deviation from the untreated population, as indicated by the black crosses. The initial 3 hours were not included because the ratio of noise to actual measurement was too high. (c) The time courses of two colonies (solid and dashed lines) are compared for a selection of cefotaxime concentration to demonstrate reproducibility. Each curve represents the average of four technical replicates from the same colony.

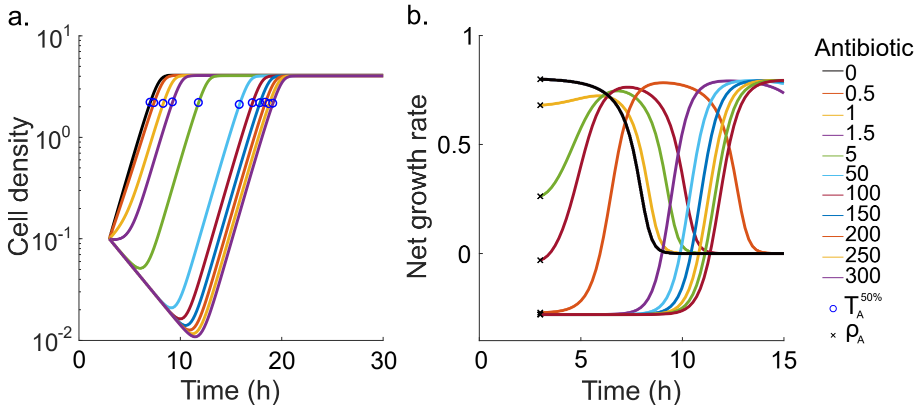

Fig. S4. Time courses and rate of change curves generated by the model. (a) Similar to the experimental data, the cell density undergoes a crash and recovery once the antibiotic concentration is high enough. With increasing antibiotic concentration, the amount of time necessary for the population to recover increases. $T^{50\%}$ was calculated as in fig. S 3a. (b) The net growth rate was used to determine ρ_A, calculated similarly to fig. S 3b.

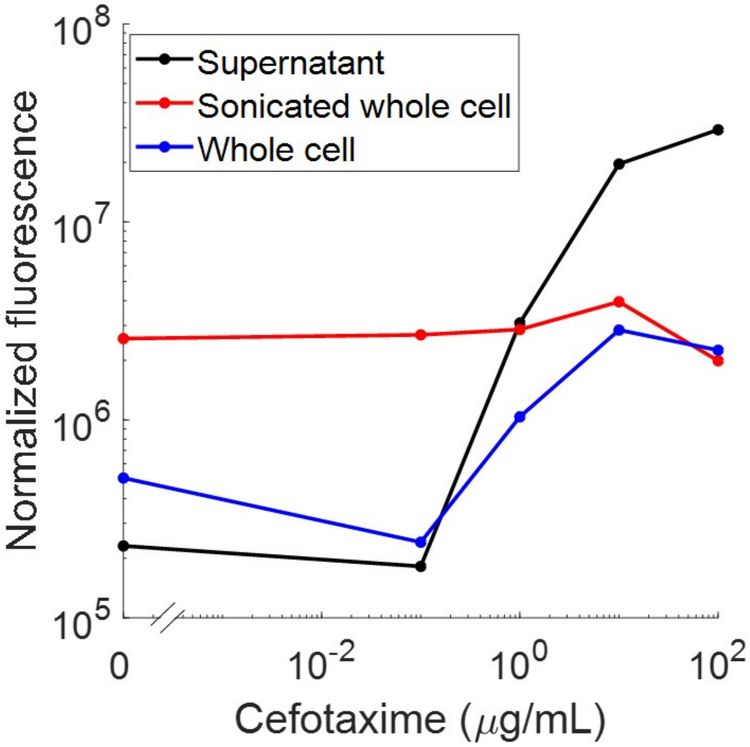

Fig. S5. Quantifying Bla activity in different components of culture. Once the cefotaxime concentration was strong enough to induce significant lysis (1 μg/mL cefotaxime), the amount of Bla activity observed in the supernatant exceeds that from the whole cells. Even if the cells are sonicated, the Bla released from the periplasm is about an order of magnitude less active than the Bla present in the supernatant.

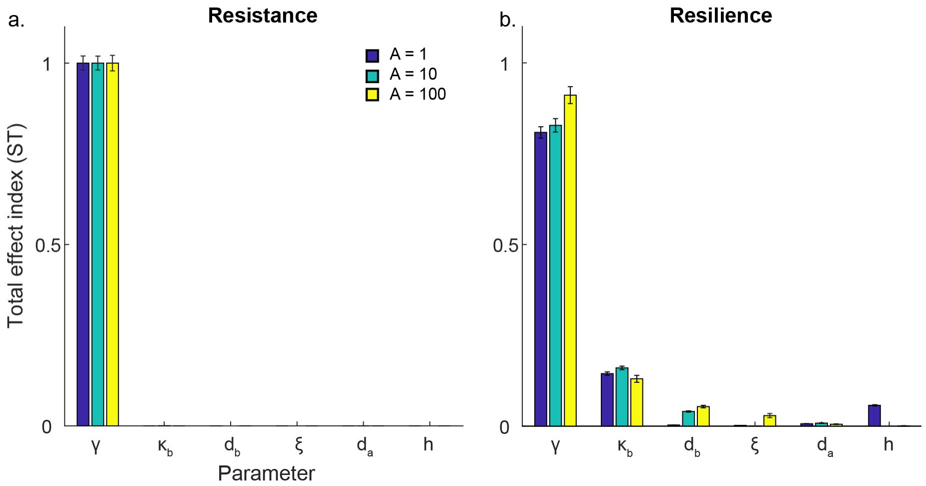

Fig. S6. Sensitivity analysis reveals parameters affecting resistance and resilience. (a) Resistance is most sensitive to the maximum lysis rate (γ) for all antibiotic concentrations tested. (b) Resilience is affected by numerous parameters. Regardless of antibiotic concentration, lysis rate has the greatest impact followed by Bla activity level (κ_b). As antibiotic concentration increases, the turnover rate of Bla (d_b) and nutrient recycling (ξ) become more influential and the Hill coefficient (h) becomes less influential. Of interest are the parameters affecting Bla activity, κ_b and d_b, because they can be targeted clinically by a Bla inhibitor.

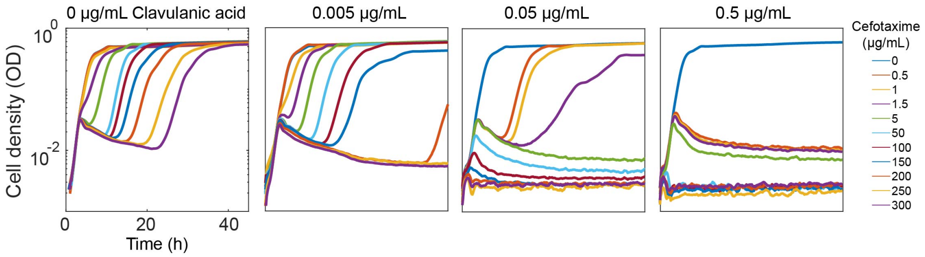

Fig. S7. Time courses showing the effects of Bla inhibition. Isolate I was exposed to the same range of cefotaxime (0 μg/mL to 300 μg/mL) in combination with clavulanic acid ranging from 0 μg/mL to 0.5 μg/mL. With increasing clavulanic acid concentrations, the isolate became increasingly sensitive to the cefotaxime as more Bla activity was inhibited.

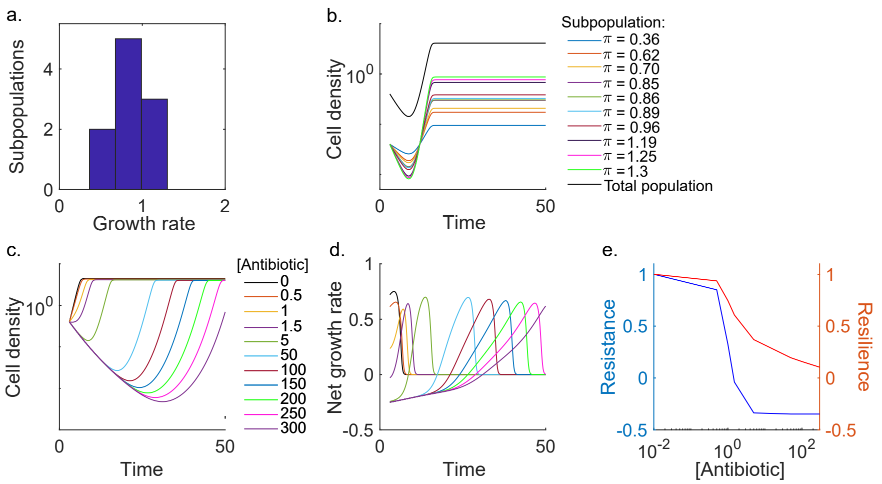

Fig. S8. Framework can be applied to heterogeneous populations. (a) Growth rates from a normal distribution were randomly generated and assigned to 10 subpopulations. (b) Time courses reveal how the growth dynamics of the different subpopulations (colored curves) determine the growth dynamics of the population as a whole. The slower growing populations experience lower degrees of lysis than their faster growing counterparts; however, given enough time, the faster growing populations are able to recover faster and ultimately make up larger portions of the population. (here, A = 5) (c-d) The resistance and resilience framework is applied the same way to the heterogeneous population. Note in (d) the slowly increasing population densities for populations treated with sufficiently high antibiotics (A >50). This is due to the averaging of subpopulations that are recovering at significantly different timescales (or not at all). ((c) and (d) share the same legend)

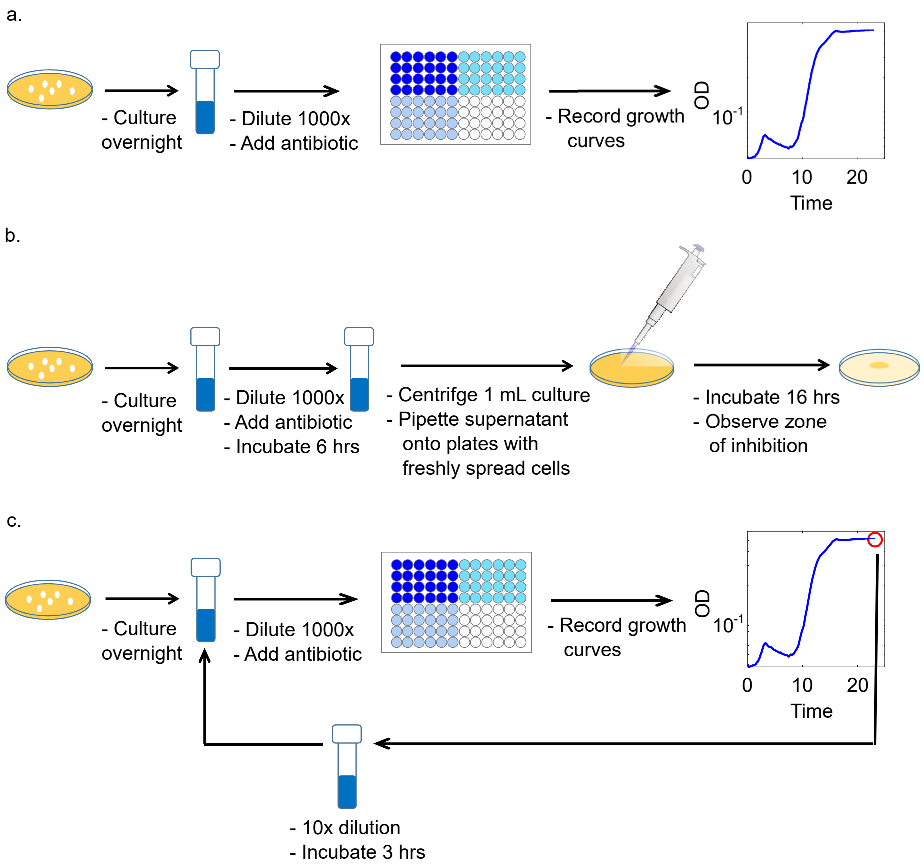

Fig. S9. Schematic for methods. (a) Time course methods: Select a single colony, inoculate it in 2 mL M9, and incubate it for 12 hours at 30 °C. Adjust the OD to 0.5, dilute the culture 1000x, and add the different concentrations of antibiotic. Set up a 96-well plate and insert into a microplate reader that collects OD measurements every 10 minutes. MATLAB was used to sort and plot the time courses. (b) Antibiotic degradation assay: A single colony of sensitive (MG1655) and ESBL-producing cells (Isolate I) were inoculated in 2 mL of M9 and incubated for 12 hours at 30 °C. Each culture was adjusted to 0.5 OD and diluted 1000x before cefotaxime was added for a final concentration of 10 or 100 µg/mL. The cultures were incubated for 6 hours at 30 °C before centrifuging to separate the supernatant. Sensitive cells were spread on fresh agar plates. Then 5 µL of the supernatant from each culture was dropped into the middle of separate plates spread with sensitive cells. The plates were then incubated for 16 hours at 37 °C. (c) Methods for re-exposing survivors to test for selection: The methods were the same as described in fig. S8a. After the first time course was completed, cells that had grown back were diluted 10x in fresh M9, grown for 3 hours at 37 °C, before repeating the methods for setting up a time course experiment.

Table S1. ESBL-producing isolates screened.

Label in paper	Isolate I	Isolate II	Isolate III	Isolate IV
Isolate ID	DICON-005	DICON-29	DICON-047	DICON-049
MicroScan ID	*K. pneumoniae*	*E. coli*	*K. oxytoca*	*E. coli*
CTX (mm)	21	21	24	28
CTX+CLA (mm)	31	33	26	32
CAZ (mm)	30	28	24	28
CAZ+CLA (mm)	32	31	24	28
MicroScan	POSITIVE	POSITIVE	POSITIVE	POSITIVE
Disk Diffusion	POSITIVE	POSITIVE	NEGATIVE	POSITIVE
Bla Variants	N/A	blaCTX-M-15<br>blaOXA-1	N/A	blaCTX-M-15<br>blaOXA-1
